# Feasibility of Computed Tomography-Guided Methods for Spatial Normalization of Dopamine Transporter Positron Emission Tomography Image

**DOI:** 10.1371/journal.pone.0132585

**Published:** 2015-07-06

**Authors:** Jin Su Kim, Hanna Cho, Jae Yong Choi, Seung Ha Lee, Young Hoon Ryu, Chul Hyoung Lyoo, Myung Sik Lee

**Affiliations:** 1 Molecular Imaging Research Center, Korea Institute Radiological and Medical Sciences, Seoul, South Korea; 2 Department of Neurology, Gangnam Severance Hospital, Yonsei University College of Medicine, Seoul, South Korea; 3 Department of Nuclear Medicine, Gangnam Severance Hospital, Yonsei University College of Medicine, Seoul, South Korea; Henry Jackson Foundation, UNITED STATES

## Abstract

**Background:**

Spatial normalization is a prerequisite step for analyzing positron emission tomography (PET) images both by using volume-of-interest (VOI) template and voxel-based analysis. Magnetic resonance (MR) or ligand-specific PET templates are currently used for spatial normalization of PET images. We used computed tomography (CT) images acquired with PET/CT scanner for the spatial normalization for [^18^F]-N-3-fluoropropyl-2-betacarboxymethoxy-3-beta-(4-iodophenyl) nortropane (FP-CIT) PET images and compared target-to-cerebellar standardized uptake value ratio (SUVR) values with those obtained from MR- or PET-guided spatial normalization method in healthy controls and patients with Parkinson’s disease (PD).

**Methods:**

We included 71 healthy controls and 56 patients with PD who underwent [^18^F]-FP-CIT PET scans with a PET/CT scanner and T1-weighted MR scans. Spatial normalization of MR images was done with a conventional spatial normalization tool (cvMR) and with DARTEL toolbox (dtMR) in statistical parametric mapping software. The CT images were modified in two ways, skull-stripping (ssCT) and intensity transformation (itCT). We normalized PET images with cvMR-, dtMR-, ssCT-, itCT-, and PET-guided methods by using specific templates for each modality and measured striatal SUVR with a VOI template. The SUVR values measured with FreeSurfer-generated VOIs (FSVOI) overlaid on original PET images were also used as a gold standard for comparison.

**Results:**

The SUVR values derived from all four structure-guided spatial normalization methods were highly correlated with those measured with FSVOI (*P* < 0.0001). Putaminal SUVR values were highly effective for discriminating PD patients from controls. However, the PET-guided method excessively overestimated striatal SUVR values in the PD patients by more than 30% in caudate and putamen, and thereby spoiled the linearity between the striatal SUVR values in all subjects and showed lower disease discrimination ability. Two CT-guided methods showed comparable capability with the MR-guided methods in separating PD patients from controls and showed better correlation between putaminal SUVR values and the parkinsonian motor severity than the PET-guided method.

**Conclusion:**

CT-guided spatial normalization methods provided reliable striatal SUVR values comparable to those obtained with MR-guided methods. CT-guided methods can be useful for analyzing dopamine transporter PET images when MR images are unavailable.

## Introduction

Dopamine transporter positron emission tomography (PET) is now popularly used in clinics to visualize the integrity of presynaptic dopaminergic nerve terminals, and a quantitation of striatal binding in PET images is important for detecting preclinical stage, monitoring disease progression, and evaluating responses to disease modifying treatment in Parkinson’s disease (PD) [1]. For the quantification of striatal binding, manually drawn striatal region-of-interest (ROI) directly on individual PET image had been used; however, this conventional ROI method is time-consuming and vulnerable to inter- and intra-operator variability [2, 3]. Moreover, as the radioligand uptake in the posterior putamen is markedly reduced even in the early stage of PD [4], it is frequently difficult to trace the putaminal margins without reference to magnetic resonance (MR) images. Therefore, a striatal volume-of-interest (VOI) template overlaid on spatially normalized PET images is required to achieve objective, user-independent quantification of striatal binding.

For spatial normalization, PET images can be directly normalized to ligand-specific PET template (PET-guided method), or indirectly to MR template by applying transformation parameters normalizing MR images to coregistered PET images (MR-guided method). While the spatial normalization of dopamine transporter PET images using ligand-specific PET template can be readily performed, it frequently causes errors in PD patients due to markedly reduced uptake in the posterior putamen. Thus, some PET studies have used summed PET images of entire or earlier time frames of dynamic PET for spatial normalization [5, 6]. The MR-guided spatial normalization method is generally more accurate than the PET-guided method and works independently from receptor density [7, 8]. Another approach based on structural image for spatial normalization is the computed tomography (CT)-guided method [9, 10]. The PET/CT scanners are now widely used, and structural CT images always accompany PET images for the attenuation correction. These CT images can be used for spatial normalization.

Spatial normalization of CT images is difficult due to great differences in intensities of the skull, brain tissue, and air. Thus, spatial normalization is mainly driven by the skull which shows extremely high intensity [9]. For better spatial normalization, Rorden and colleagues developed a CT-guided spatial normalization method by using intensity-transformed CT (itCT) images to reduce intensity contrast between the skull and brain tissue [9]. Skull-stripping to remove the skull and surrounding soft tissue is also helpful for reducing errors in the spatial normalization of CT images, as well as MR images [9–13], but there was no reliable method for skull-stripping of CT images.

We recently developed fully automated skull-stripping method for CT images based on tissue probabilistic templates, and showed the feasibility of CT-guided spatial normalization for [^18^F]-fluorodeoxyglucose (FDG) PET imaging [14]. In this study, we applied our CT-guided spatial normalization technique for the spatial normalization of [^18^F]-N-3–fluoropropyl-2-b-carboxymethoxy-3-b-(4-iodophenyl) nortropane (FP-CIT) PET images in PD patients and sought to determine its feasibility by comparing the striatal binding derived from the itCT-, PET-, and two MR-guided spatial normalization methods.

## Materials and Methods

### Subjects

We included 56 consecutive PD patients (M: F = 25: 31, mean age = 61.1 ± 12.4 years) who met the UK brain bank criteria for the clinical diagnosis of PD [15] and who underwent [^18^F]-FP-CIT PET and brain MRI scans for diagnostic work-up over a two year period beginning in May 2011. In 56 PD patients, 39 patients had never been treated for PD, and the remaining 17 patients were on anti-parkinsonian medications. Right side limbs were clinically more affected in 22 patients, while the remaining 34 patients were affected more on the left side. The mean disease duration was 32.1 ± 33.3 months; the mean unified Parkinson’s disease rating scale (UPDRS) motor score was 28.0 ± 8.1; and the mean Hoen & Yahr stage was 2.3 ± 0.3. We also included 71 age-matched healthy controls (M: F = 32: 39, mean age = 59.7 ± 10.3 years) who had no history of neurological illness and no abnormal sign on neurological examination. The mean ages of the healthy controls and PD patients were not different. This study was approved by Institutional Review Board (IRB) of Gangnam Severance Hospital and written informed consent was obtained from all healthy control subjects. As the PET and MR images of the PD patients were obtained during the process of diagnostic work-up at a tertiary hospital, the IRB approved for the use of their image data and minimal clinical information without the need to obtain consent due to the retrospective nature of the study.

### Acquisition of PET and MR images

We acquired brain CT and [^18^F]-FP-CIT PET scans using a Biograph 40 TruePoint PET/CT scanner (Siemens Medical Solutions; Malvern, PA, USA). At 3 hours after the intravenous injection of 214.4 ± 35.4 MBq (212.1 ± 31.2 MBq for healthy controls and 217.4 ± 40.2 MBq for PD patients) of [^18^F]-FP-CIT, brain CT scans (120 keV, 180 mAs, and 2 mm of slice thickness) were acquired for attenuation correction, followed by 10 minutes of [^18^F]-FP-CIT emission PET scan. A head holder was applied to minimize head motion during the scan. 3D CT and PET images were reconstructed with a 512 × 512 × 110 matrix using ordered-subsets expectation maximization (OSEM) algorithm (iteration = 6 and subset = 16). The voxel size was 0.668 × 0.668 × 2 mm. In all subjects, axial T1-weighted brain MR images were also obtained with 3D-spoiled gradient-recalled sequences (3D-SPGR sequences; repetition time = 6.8 ms, echo time = 1.6 to 11.0 ms, flip angle = 20°, 512 × 512 matrix, voxel spacing 0.469 × 0.469 × 1 mm) in 3.0 Tesla scanner (Signa EXCITE, GE Medical Systems, Milwaukee, WI).

### Image processing

We used statistical parametric mapping 8 software (SPM8; Wellcome Trust Centre for Neuroimaging, London, UK) implemented in MATLAB 7.1 (MathWorks, Natick, MA) for coregistration and spatial normalization of images and voxel-based comparison. We also used in-house MATLAB programs for simple arithmetic operation of images and measuring regional uptake values.

#### a) Creation of CT templates

Using image data from 71 healthy controls, we created two types of CT templates for spatial normalization; skull-stripped CT (ssCT) template [14] and an intensity-transformed CT (itCT) template [9].

**Fig 1:**
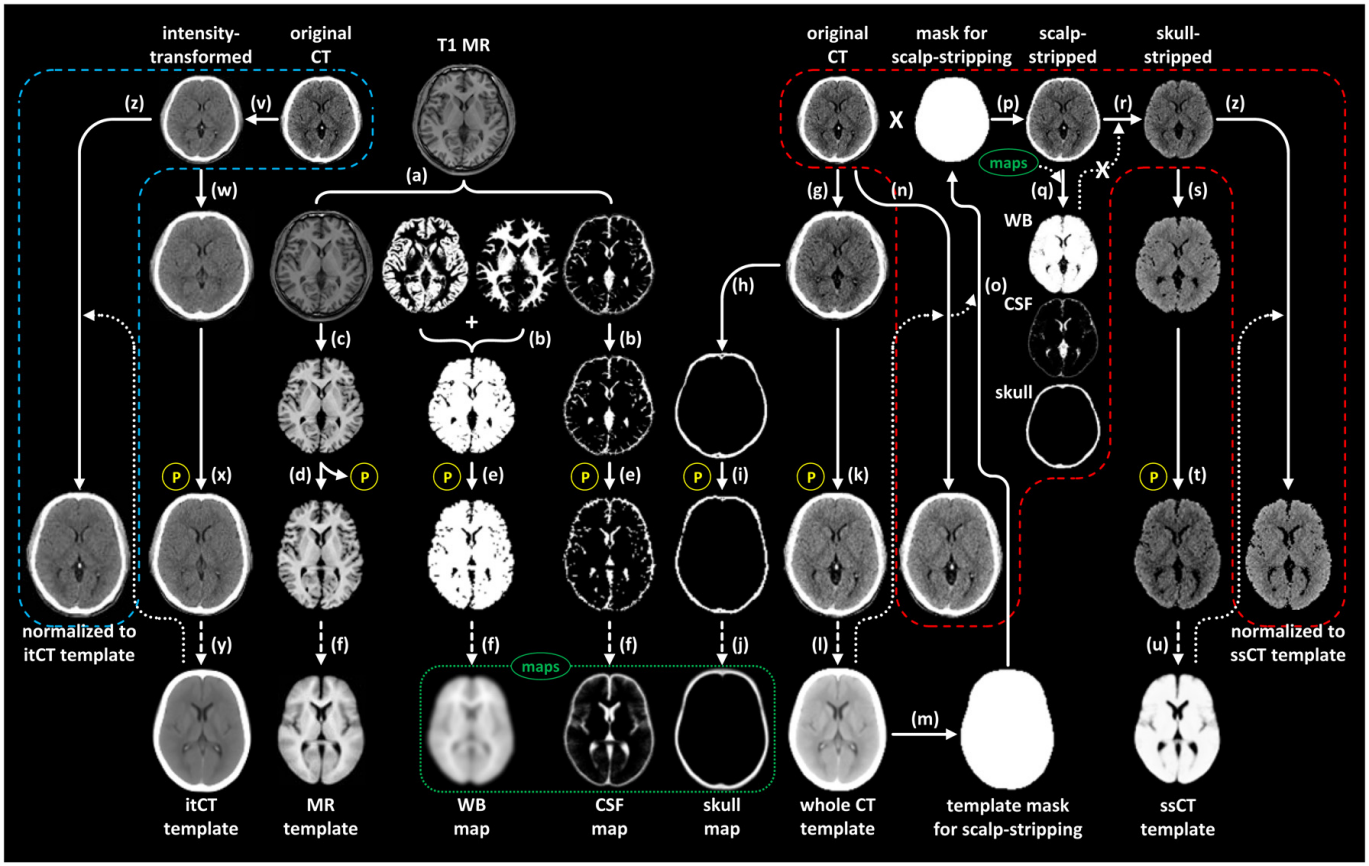
Image processing steps for acquiring skull-stripped CT (ssCT) and intensity-transformed CT (itCT) templates. (a) Inhomogeneity correction and segmentation of MR, (b) creation of whole brain and CSF masks, (c) skull-stripping of inhomogeneity-corrected MR, (d) spatial normalization of skull-stripped MR to MNI template for skull-stripped MR, (e) spatial normalization of masks by applying normalization parameter, (f) creation of probabilistic maps for whole brain and CSF by averaging tissue masks, (g) coregistration of CT to inhomogeneity-corrected MR, (h) creation of skull mask, (i) spatial normalization of skull mask, (j) creation of probabilistic map for skull, (k) spatial normalization of coregistered CT, (l) creation of whole CT template by averaging, (m) creation of template mask for scalp-stripping, (n) spatial normalization of CT to whole CT template, (o) inverse normalization of template mask for scalp-stripping to create individual mask for scalp-stripping, (p) creation of scalp-stripped CT by applying mask, (q) segmentation of scalp-stripped CT into three tissue type by using probabilistic maps, (r) creation of ssCT by applying mask for whole brain segment, (s) coregistration of ssCT to MR by applying parameter coregistering original CT to MR, (t) spatial normalization of coregistered ssCT, (u) creation of ssCT template by averaging, (v) intensity transformation of original CT, (w) coregistration of itCT to MR, (x) spatial normalization of coregistered itCT, (y) creation of itCT template by averaging, and (z) spatial normalization of individual ssCT and itCT to specific CT templates. The processing steps inside the red (ssCT) and blue (itCT) dashed lines represent the image processing steps required for the spatial normalization of CT images with created CT templates.

For creation of the ssCT template, the intensity of CT images was first linearly transformed by adding minimum voxel values to eliminate negative voxel values. The skull-stripped MR images were obtained by merging the gray and white matter segments of inhomogeneity-corrected T1-weighted MR images (Fig 1a–1c) and normalized to the skull-stripped Montreal Neurological Institute (MNI) 152 MR template to derive spatial normalization parameters for MR images (Fig 1d). Probabilistic maps for whole brain and cerebrospinal fluid (CSF) were created by averaging the spatially normalized masks of each MR segment (Fig 1e and 1f). Also, the probabilistic map for skull was created with the spatially normalized masks of skull segments of CT images (Fig 1g–1j). The CT images coregistered to individual MR images were spatially normalized with the spatial normalization parameters for MR images (Fig 1g–1k), and we created a temporary whole CT template and a scalp-stripping mask, including skull, whole brain, CSF and soft tissue below the convexity of skull (Fig 1l and 1m). All CT images were spatially normalized to this whole CT template (Fig 1n) and scalp-stripped by applying inversely normalized scalp-stripping mask images (Fig 1o and 1p). Scalp-stripped CT images were segmented into skull, whole brain, and CSF using the probabilistic maps for each tissue type in SPM segmentation tool (Fig 1q), and then ssCT images were obtained by applying a binary whole brain mask to the original CT images (Fig 1r). Finally, the ssCT template was created by averaging all ssCT images normalized to the MNI template space with spatial normalization parameters for MR images (Fig 1s–1u). Detailed image processing steps for acquiring the ssCT template were described in our previous paper [14].

For creation of the itCT template, we first transformed the intensity of native CT images encoded with Hounsfield units into three predefined levels in order to obtain itCT images (Fig 1v) [9]. Voxel values equal to or less than -100 were linearly converted to greater values by adding 1000, and those greater than 100 were converted to values greater than 3100 by adding 3000. The voxel values between -100 and 100 were linearly rescaled to a range between 900 and 3100. Thereby, most of the negative voxel values in air were eliminated, and the intensity range representing brain tissue and CSF was increased. After the coregistration of itCT images to inhomogeneity-corrected individual MR images (Fig 1w), itCT images were normalized to the MNI space by applying spatial normalization parameters for MR images (Fig 1x). Finally, the itCT template image was created by averaging all spatially normalized itCT images of 71 healthy controls (Fig 1y).

#### b) Creation of MR templates and ligand-specific PET template

We used two types of MR-guided spatial normalization methods: 1) MR-guided spatial normalization with a conventional spatial normalization tool in SPM8 (cvMR) and 2) MR-guided spatial normalization with diffeomorphic anatomical registration using exponentiated lie algebra (DARTEL) toolbox in SPM8 (dtMR) [16]. For the cvMR-guided spatial normalization, a skull-stripped MR template image was created by averaging all skull-stripped and spatially normalized MR images of 71 healthy controls (Fig 1f). For the dtMR-guided spatial normalization, MR images were segmented into gray (GM) and white matter (WM), and then segmented GM/WM images were imported to 1.5 mm isovoxel space by applying normalization parameters obtained from the segmentation step. An initial template was created by averaging imported GM/WM images of all controls and PD patients, and then a deformation transforming initial template to individual subject’s GM/WM was acquired. Inverse of this deformation was applied to GM/WM images, and then averaged to create next template. After the iteration of this deformation steps for six times, final GM/WM template was created.

All [^18^F]-FP-CIT PET images were coregistered to inhomogeneity-corrected individual MR images and spatially normalized to MNI template space with the parameter normalizing individual skull-stripped MR images in the conventional normalization of MR images. A ligand-specific PET template image was created by averaging all spatially normalized PET images.

#### c) Spatial normalization of PET images

We obtained spatially normalized [^18^F]-FP-CIT PET images using five different spatial normalization methods: cvMR-, dtMR-, ssCT-, itCT-, and PET-guided spatial normalization. 1) For the cvMR-guided spatial normalization, the PET images were coregistered to individual MR images and spatially normalized to the MNI space with the parameter normalizing skull-stripped MR. 2) For the dtMR-guided spatial normalization, the PET images coregistered to the MR images were spatially normalized to the MNI space by applying the subject-specific DARTEL flow fields. 3) For the ssCT- guided spatial normalization, ssCT images were obtained by scalp-stripping and segmentation of CT images, and then individual PET images were spatially normalized with the parameter normalizing individual ssCT images to the ssCT template (Fig 1n–1r and 1z). 4) For the itCT-guided spatial normalization, the PET images were spatially normalized with the parameter normalizing individual itCT images to the itCT template (Fig 1v and 1z). 5) For the PET-guided spatial normalization, individual PET images were directly spatially normalized to the ligand-specific PET template.

#### d) Creation of subject-specific volume of interest (VOI)

We used subject-specific striatal VOIs derived from FreeSurfer 5.1 (Massachusetts General Hospital, Harvard Medical School; http://surfer.nmr.mgh.harvard.edu) as a gold standard for comparing striatal [^18^F]-FP-CIT binding values derived from five different spatial normalization methods. T1-weighted MR images were resliced to 1 mm isovoxel space, corrected for inhomogeneity, skull-stripped, and segmented into gray and white matter. And then, subcortical structures were segmented and labelled by a probabilistic registration technique [17]. We chose five segmented subcortical segments of the caudate and putamen for each side, as well as that of the cerebellum. These subcortical segments were coregistered to original PET images by using the parameter coregistering inhomogeneity-corrected MR images created by FreeSurfer to CT images in native space, and finally, FreeSurfer-generated specific VOIs (FSVOI) for the caudate, putamen and cerebellum were created. For the striatal subregional analysis, a striatal subregional VOI template was non-linearly transformed to FSVOI by applying the inverse of the normalization parameter transforming the MR images to the MR template [18, 19]. Thereby, FSVOI was further parcellated into five striatal subregions (anterior and posterior caudate, anterior and posterior putamen, and ventral striatum).

#### e) Creation of VOI templates

To measure striatal and cerebellar binding values in spatially normalized PET images, we created VOI templates for the caudate, putamen, and cerebellum. The striatal and cerebellar segments obtained from subcortical parcellation by FreeSurfer were spatially normalized to the skull-stripped MR template within 1 mm sized isovoxel space. Normalized segments of each region were binarized, and the probabilistic maps for each region were created by averaging binary segments of all 71 healthy controls. We chose threshold 0.8 to create masks for each region, and finally created a VOI template within the 2 mm sized isovoxel space corresponding to the conventional MNI template. For the striatal subregional analysis, we parcellated the striatal VOI template into five striatal subregions.

### Estimation of standardized uptake value ratio (SUVR)

Using the VOI template, we measured regional SUV values of the cerebellum and each side of the caudate and putamen in the PET images spatially normalized in five different methods. Regional SUV values measured by using subject-specific FSVOI were considered to be a gold standard. Mean SUVR values of both sides of the caudate and putamen were calculated as follows:

$${\text{SUVR}}_{\text{target}}{\text{= SUV}}_{\text{target}}{\text{ / SUV}}_{\text{cerebellum}}$$

### Statistical analysis

We used Prism 5 (GraphPad Software, Inc., La Jolla, CA, USA) for the statistical analyses, and performed linear regression analysis between the SUVR values measured with specific FSVOI on original PET images and the VOI template on the PET images spatially normalized by each normalization method. Also, we checked the correlation between the striatal SUVR values and the clinical severity of PD. Independent t-test was used for the group comparison and the receiver-operator characteristic (ROC) curve analysis was used for the assessment of disease discrimination ability of these different methods. For each spatial normalization method, we also compared SUVR images between the healthy controls and PD patients using the voxel-based independent t-test of SPM8.

## Results

**Fig 2:**
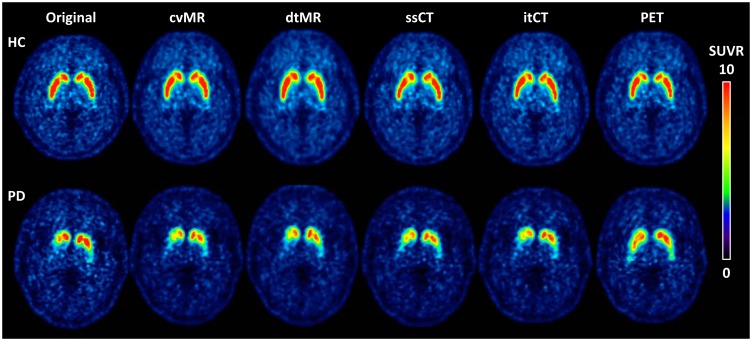
Examples of spatial normalization by five different spatial normalization methods in a healthy control and a PD patient. Four structure-guided spatial normalization methods work well for normalization of PET images, and the PET-guided method also works well in healthy control. However, in PD patient the PET-guided method stretches anterior putamen posteriorly to fill the posterior putamen during the non-linear spatial normalization. Color bars are scaled in standardized uptake value ratio (SUVR). Abbreviations: cvMR = MR-guided spatial normalization with conventional normalization tool, dtMR = MR-guided spatial normalization with DARTEL toolbox, ssCT = skull-stripped CT-guided spatial normalization, itCT = intensity transformed CT-guided spatial normalization, PET = PET-guided spatial normalization.

All structure-guided spatial normalization methods (cvMR-, dtMR-, ssCT- and itCT-guided methods) were similarly effective for normalizing [^18^F]-FP-CIT PET images of healthy controls and PD patients in visual inspection of the spatially normalized images. However, in contrast to appropriate spatial normalization results achieved by the PET-guided method in healthy controls, in PD patients showing markedly reduced [^18^F]-FP-CIT uptake in the posterior putamen, PET-guided spatial normalization erroneously stretched remaining anterior putamen to fill the defective posterior putamen (Fig 2).

**Fig 3:**
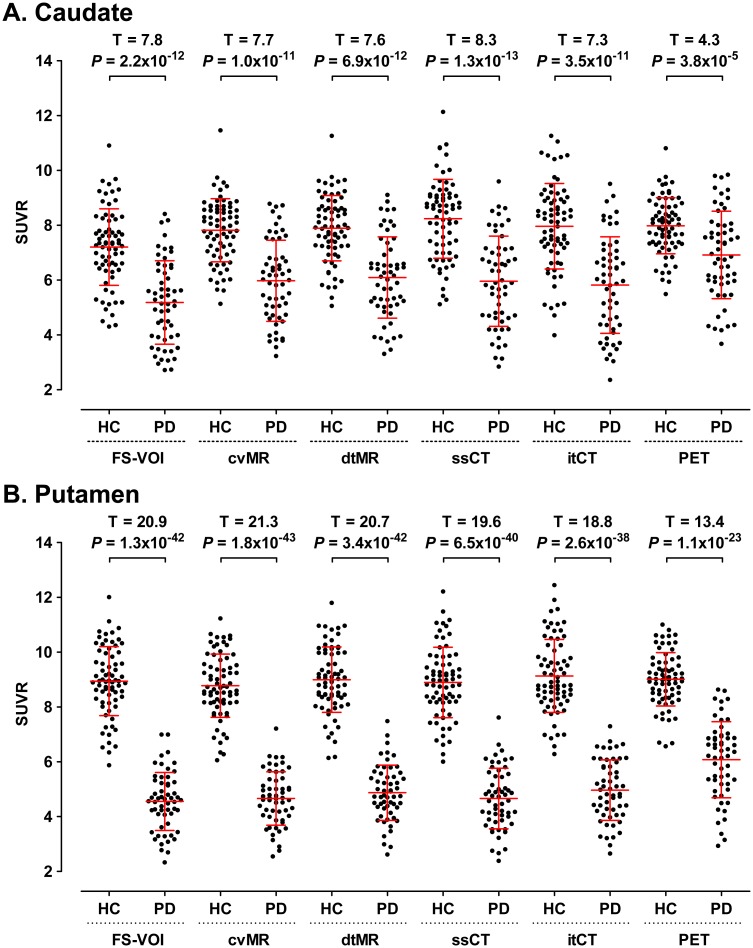
Striatal standardized uptake value ratio (SUVR) values (A: caudate, B: putamen) of healthy controls (HC) and Parkinson’s disease (PD) patients. The horizontal red bars represent mean and standard deviation (SD). T- and *P*-values for comparing two groups with independent t-test are presented on the top of the graphs. Abbreviations: FSVOI = FreeSurfer-generated volume of interest, cvMR = MR-guided spatial normalization with conventional normalization tool, dtMR = MR-guided spatial normalization with DARTEL toolbox, ssCT = skull-stripped CT-guided spatial normalization, itCT = intensity transformed CT-guided spatial normalization, PET = PET-guided spatial normalization.

Using subject-specific striatal FSVOIs, we measured SUVR values of the caudate (mean SUVR in HC = 7.2 ± 1.4 and that in PD = 5.2 ± 1.5) and putamen (mean SUVR in HC = 8.9 ± 1.3 and that in PD = 4.6 ± 1.1) for the comparison with those measured with striatal template VOIs overlaid on the PET images normalized by five different spatial normalization methods (Table 1). Although there were highly significant differences in caudate and putaminal SUVR values between the controls and PD patients (*P* < 10^-4^ for caudate, *P* < 10^-22^ for putamen), there was greater range of overlapping SUVR values derived from the PET-guided method between the two diagnostic groups (Fig 3). The cvMR-, dtMR-, ssCT-, and itCT-guided methods resulted in overestimation of caudate SUVR values by 8.5 to 17.6%, compared to those measured by FSVOI. For the putamen, the bias was much smaller for those four structure-guided methods (-1.9 to 9.0%). The biases of SUVR values derived from the PET-guided method were almost similar to those derived from structure-guided methods in the controls (caudate: 10.8%, putamen: 0.8%), but markedly greater in PD patients (caudate: 33.5%, putamen: 33.4%). These overestimation biases of the PET-guided method in PD patients were greater in the regions contralateral to the clinically worse side than in the regions contralateral to the clinically better side, most prominent in the anterior putamen (57.6%) and lowest in the ventral striatum (17.1%) (Table 1).

**Table 1:** Striatal SUVR values of healthy controls (HC) and Parkinson’s disease (PD) patients.

		FSVOI	cvMR		dtMR		ssCT		itCT		PT	
		mean ± SD	mean ± SD	bias	mean ± SD	bias	mean ± SD	bias	mean ± SD	bias	mean ± SD	bias
**Caudate**												
** HC**	**both**	7.2 **±** 1.4	7.8 **±** 1.1	8.5	7.9 **±** 1.2	9.6	8.2 **±** 1.4	14.3	8.0 **±** 1.6	10.5	8.0 **±** 1.0	10.8
** PD**	**both**	5.2 **±** 1.5	6.0 **±** 1.5	15.2	6.1 **±** 1.5	17.6	6.0 **±** 1.6	15.0	5.8 **±** 1.8	12.3	6.9 **±** 1.6	33.5
	**worse**	5.0 **±** 1.6	5.8 **±** 1.4	16.3	5.9 **±** 1.5	19.6	5.8 **±** 1.6	16.8	5.6 **±** 1.8	12.9	6.7 **±** 1.6	36.2
	**better**	5.4 **±** 1.6	6.2 **±** 1.6	14.6	6.3 **±** 1.6	16.1	6.1 **±** 1.8	13.5	6.0 **±** 2.0	12.1	7.1 **±** 1.6	31.3
**Anterior caudate**												
** HC**	**both**	7.9 **±** 1.5	8.3 **±** 1.3	4.8	8.5 **±** 1.3	8.2	8.8 **±** 1.6	11.8	8.5 **±** 1.8	7.9	8.5 **±** 1.2	7.5
** PD**	**both**	5.7 **±** 1.8	6.3 **±** 1.6	10.7	6.6 **±** 1.7	16.4	6.3 **±** 1.8	11.7	6.2 **±** 2.0	9.8	7.4 **±** 1.8	30.5
	**worse**	5.4 **±** 1.8	6.0 **±** 1.6	11.6	6.4 **±** 1.7	18.4	6.1 **±** 1.8	13.7	6.0 **±** 2.0	10.6	7.2 **±** 1.8	33.8
	**better**	5.9 **±** 1.8	6.5 **±** 1.7	10.3	6.8 **±** 1.7	15.1	6.5 **±** 2.0	10.4	6.5 **±** 2.2	9.6	7.6 **±** 1.8	28.0
**Posterior caudate**												
** HC**	**both**	5.5 **±** 1.4	5.6 **±** 0.9	1.3	4.7 **±** 1.1	-15.1	6.2 **±** 1.3	13.1	5.8 **±** 1.6	5.2	5.5 **±** 0.7	-0.7
** PD**	**both**	3.8 **±** 1.1	4.3 **±** 1.1	14.2	3.3 **±** 0.9	-12.0	4.5 **±** 1.3	18.9	4.0 **±** 1.6	6.4	5.0 **±** 1.3	31.1
	**worse**	3.5 **±** 1.2	4.2 **±** 1.0	19.6	3.3 **±** 0.9	-7.5	4.4 **±** 1.3	25.3	3.8 **±** 1.7	8.1	4.8 **±** 1.4	34.9
	**better**	3.9 **±** 1.3	4.5 **±** 1.2	13.6	3.4 **±** 1.0	-12.8	4.6 **±** 1.4	17.2	4.2 **±** 1.7	7.2	5.1 **±** 1.3	30.2
**Putamen**												
** HC**	**both**	8.9 **±** 1.3	8.8 **±** 1.2	-1.9	9.0 **±** 1.2	0.6	8.9 **±** 1.3	-0.5	9.1 **±** 1.3	2.1	9.0 **±** 1.0	0.8
** PD**	**both**	4.6 **±** 1.1	4.7 **±** 1.0	2.3	4.9 **±** 1.0	7.0	4.7 **±** 1.1	2.3	5.0 **±** 1.1	9.0	6.1 **±** 1.4	33.4
	**worse**	4.2 **±** 1.0	4.2 **±** 0.9	1.1	4.4 **±** 0.9	5.7	4.3 **±** 1.0	2.4	4.6 **±** 1.0	10.7	5.9 **±** 1.4	40.3
	**better**	4.9 **±** 1.4	5.1 **±** 1.2	3.0	5.3 **±** 1.2	7.6	5.0 **±** 1.4	1.9	5.3 **±** 1.4	7.2	6.3 **±** 1.4	27.1
**Anterior putamen**												
** HC**	**both**	9.2 **±** 1.4	10.1 **±** 1.4	9.4	10.2 **±** 1.4	10.7	10.0 **±** 1.5	8.6	10.1 **±** 1.4	9.0	10.4 **±** 1.2	12.1
** PD**	**both**	4.6 **±** 1.3	5.2 **±** 1.4	13.5	5.4 **±** 1.4	18.1	5.1 **±** 1.5	12.3	5.6 **±** 1.5	23.1	7.2 **±** 1.8	57.6
	**worse**	4.1 **±** 1.2	4.6 **±** 1.2	12.5	4.8 **±** 1.3	17.3	4.7 **±** 1.4	13.2	5.2 **±** 1.4	25.9	7.0 **±** 1.8	68.9
	**better**	5.0 **±** 1.7	5.8 **±** 1.7	14.8	6.0 **±** 1.7	19.3	5.6 **±** 2.0	12.0	6.1 **±** 1.9	21.3	7.5 **±** 1.9	48.8
**Posterior putamen**												
** HC**	**both**	8.9 **±** 1.4	8.6 **±** 1.2	-3.7	8.8 **±** 1.3	-1.5	8.7 **±** 1.5	-3.0	9.1 **±** 1.5	2.0	9.0 **±** 1.1	0.3
** PD**	**both**	3.4 **±** 0.9	3.5 **±** 0.8	5.0	3.7 **±** 0.9	10.5	3.6 **±** 1.0	5.7	3.8 **±** 0.9	12.2	4.9 **±** 1.3	46.4
	**worse**	3.0 **±** 1.0	3.2 **±** 0.8	5.4	3.3 **±** 0.9	10.0	3.2 **±** 0.9	7.0	3.4 **±** 0.9	14.8	4.8 **±** 1.3	59.9
	**better**	3.8 **±** 1.2	3.9 **±** 1.1	3.6	4.2 **±** 1.1	9.8	3.9 **±** 1.3	3.6	4.1 **±** 1.1	8.8	5.1 **±** 1.3	34.2
**Ventral striatum**												
** HC**	**both**	8.5 **±** 1.1	8.0 **±** 1.0	-5.7	8.4 **±** 1.0	-1.5	8.2 **±** 1.1	-3.3	8.3 **±** 1.3	-2.3	8.1 **±** 0.8	-4.4
** PD**	**both**	5.9 **±** 1.5	6.2 **±** 1.3	4.0	6.5 **±** 1.4	9.4	6.0 **±** 1.4	1.9	6.2 **±** 1.5	5.0	6.9 **±** 1.4	17.1
	**worse**	5.7 **±** 1.4	5.9 **±** 1.3	3.2	6.2 **±** 1.3	9.5	5.8 **±** 1.4	2.2	6.1 **±** 1.5	6.5	6.7 **±** 1.4	18.7
	**better**	6.2 **±** 1.6	6.4 **±** 1.5	4.2	6.7 **±** 1.5	8.9	6.2 **±** 1.6	1.1	6.4 **±** 1.6	3.2	7.1 **±** 1.4	15.1

Biases were calculated as the % difference between the SUVR values derived from each spatial normalization method and those measured with FSVOI. For PD patients, data are presented with the SUVR values of combined region of both sides (both), those of the regions contralateral to the clinically worse side (worse), and those of the regions contralateral to the clinically better side (better). Abbreviations: FSVOI = FreeSurfer-generated volume of interest, cvMR = MR-guided spatial normalization with conventional tool, dtMR = MR-guided spatial normalization with DARTEL toolbox, ssCT = skull-stripped CT-guided spatial normalization, itCT = intensity transformed CT-guided spatial normalization, PET = PET-guided spatial normalization

**Fig 4:**
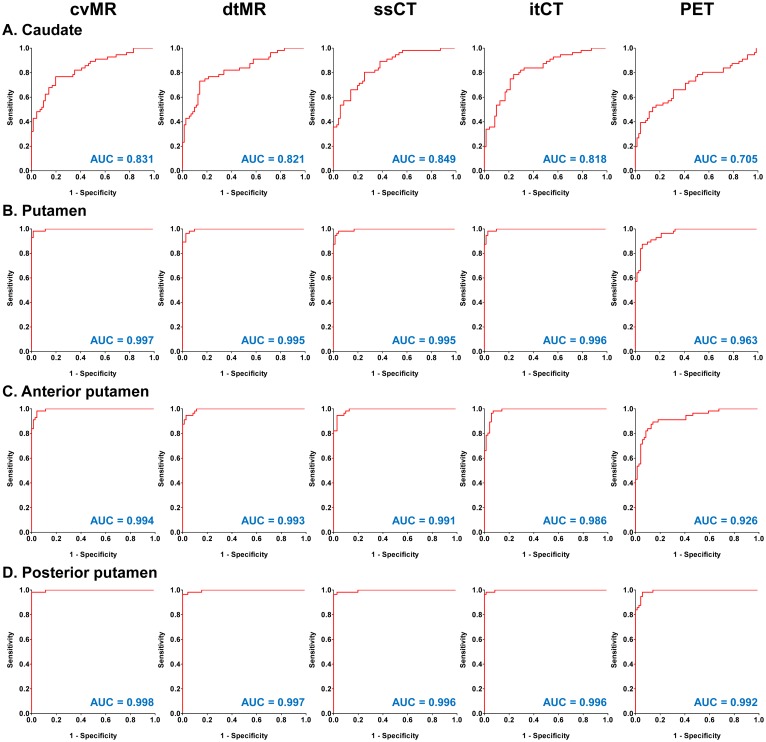
Receiver-operator characteristic (ROC) curve analysis of striatal SUVR values derived from five different spatial normalization methods for the separation of controls and Parkinson’s disease (PD) patients. The area under the curve (AUC) values are presented in the right lower corner of each graph. Putaminal SUVR values derived from all five spatial normalization methods very effectively discriminated PD patients. Both CT-guided methods show similar performance to both MR-guided methods. However, the PET-guided method was less effective than the other four structure-guided spatial normalization methods. Abbreviations: cvMR = MR-guided spatial normalization with conventional normalization tool, dtMR = MR-guided spatial normalization with DARTEL toolbox, ssCT = skull-stripped CT-guided spatial normalization, itCT = intensity transformed CT-guided spatial normalization, PET = PET-guided spatial normalization.

The ROC curve analysis of these SUVR values for the assessment of the discrimination ability between two diagnostic groups showed that putaminal SUVR values derived from all four structure-guided spatial normalization methods were highly effective for discriminating PD patients from controls (Fig 4). However, although the PET-guided spatial normalization method was also effective, the area under the ROC curve (AUC) value was lowest among the five spatial normalization methods. In contrast to very small difference in posterior putaminal AUC values between the PET- and the other four structure-guided spatial normalization methods (Fig 4D), the difference in AUC values for the anterior putamen were greater than that for the posterior putamen (Fig 4C).

**Fig 5:**
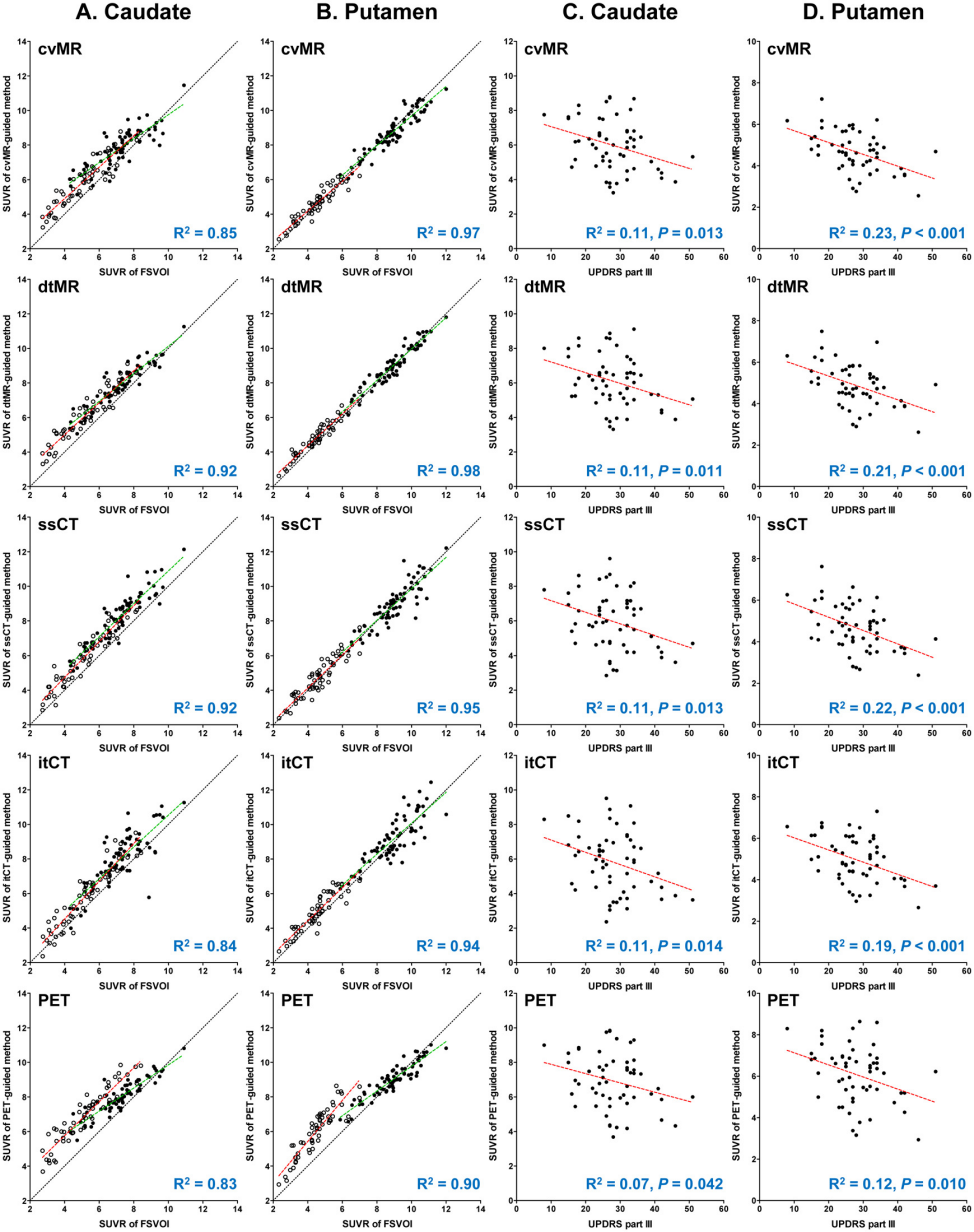
Regression analysis between the standardized uptake value ratio (SUVR) values measured with individual volume of interest (FSVOI) and those derived from five different spatial normalization methods (A and B), and correlation between the regional SUVR values and the clinical severity scores (C and D). In A and B, regression lines for each diagnostic group are presented with green (healthy controls; HC) and red (Parkinson’s disease; PD) dotted lines. Closed (HC) and open (PD) circles represent regional SUVR values of individual subjects. The R^2^ values for regression analysis in all subjects are presented in the right lower corner of each graph. The PET-guided spatial normalization method excessively overestimates striatal SUVR values in PD patients, and results in higher discrepancy in the regression line between the controls and PD patients. In C and D, closed circles represent individual striatal SUVR values and the red lines represent regression lines. The SUVR values derived from all five spatial normalization methods are correlated with clinical severity of PD, but the statistical power is lowest for the PET-guided method. Abbreviations: FSVOI = FreeSurfer-generated volume of interest, cvMR = MR-guided spatial normalization with conventional normalization tool, dtMR = MR-guided spatial normalization with DARTEL toolbox, ssCT = skull-stripped CT-guided spatial normalization, itCT = intensity transformed CT-guided spatial normalization, PET = PET-guided spatial normalization.

Similarly, in PD and control subjects combined, SUVR values derived from all four structure-guided methods well correlated with those measured with FSVOI (*P* < 0.0001; R^2^ for caudate > 0.84, R^2^ for putamen > 0.94). However, the PET-guided method excessively overestimated striatal SUVR values in the PD patients, and thereby spoiled the linearity between the striatal SUVR values in all subjects (Fig 5A and 5B). The striatal regional SUVR values derived from all four structure-guided spatial normalization methods correlated well with those measured by subregional FSVOI. However, except ventral striatum in which binding is relatively preserved, the correlation between the subregional SUVR values derived from the PET-guided method and those measured by subregional FSVOI was the lowest (S3 Fig).

The caudate and putaminal SUVR values derived from the five normalization methods were correlated with clinical severity measured with UPDRS motor score in PD patients. However, the statistical significance for the PET-guided method was lowest among the five methods (caudate: R^2^ = 0.07, *P* = 0.042 and putamen: R^2^ = 0.12, *P* = 0.01) (Fig 5C and 5D). Although two CT-guided spatial normalization methods (ssCT- and itCT-guided) showed almost similar results for the correlation with the SUVR values measured with FSVOI (ssCT: caudate R^2^ = 0.92, putamen R^2^ = 0.95 *vs*. itCT: caudate R^2^ = 0.84, putamen R^2^ = 0.94) and with clinical severity (ssCT: caudate R^2^ = 0.11, putamen R^2^ = 0.22 *vs*. itCT: caudate R^2^ = 0.11, putamen R^2^ = 0.19), the ssCT-guided method showed slightly better correlations (Fig 5) as well as group differences and discrimination (Figs 3 and 4).

**Fig 6:**
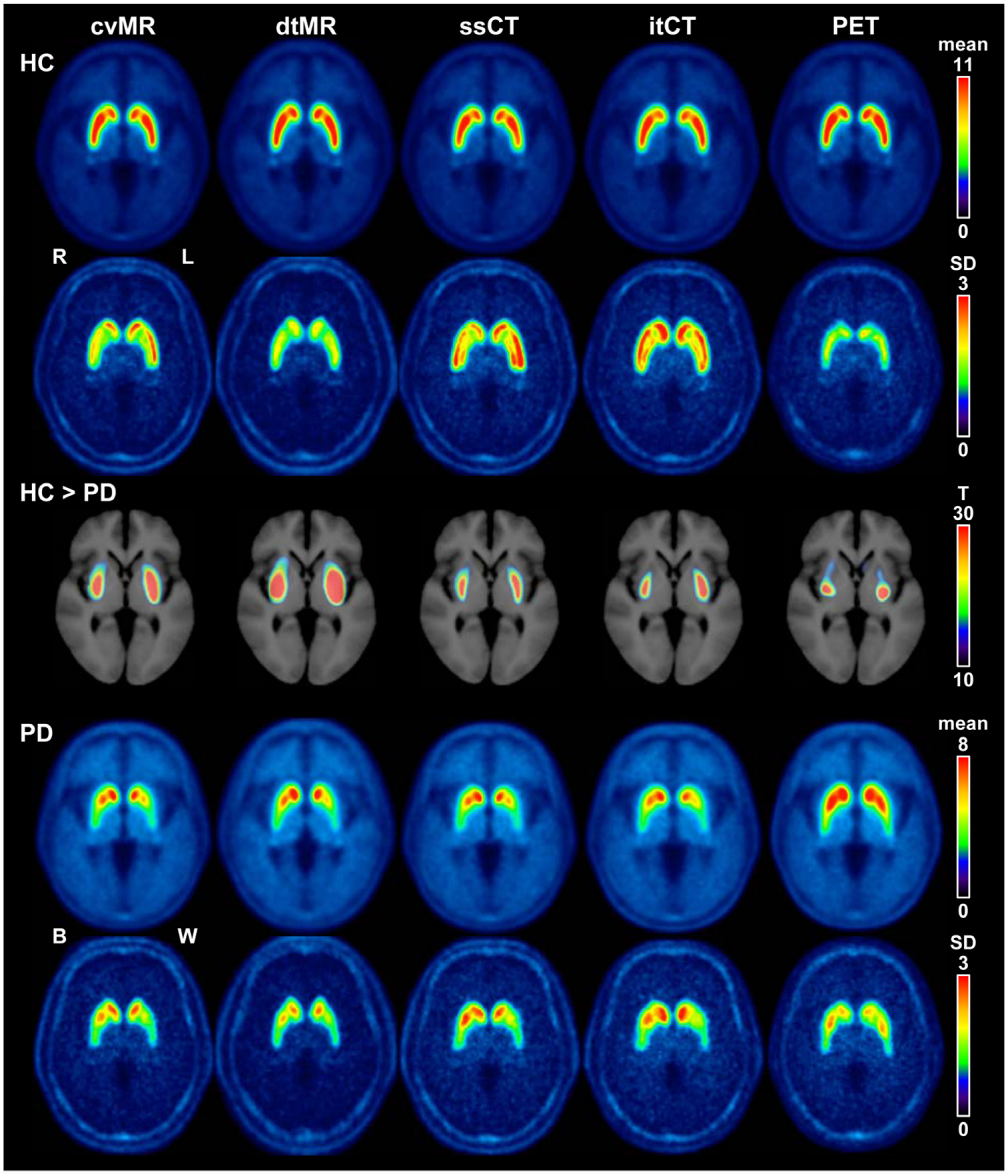
Mean and standard deviation (SD) images of each spatial normalization method and the results of voxel-based comparison between the healthy controls (HC) and Parkinson’s disease (PD) patients. For PD patients images were flipped to locate more affected side (contralateral to the clinically worse side; W) on the left (L) and the less affected side (contralateral to the clinically better side; B) on the right (R). The mean images of controls and PD patients derived from four structure-guided spatial normalization methods are similar. However, the caudate and anterior part of the putamen of PD patients were overestimated, and thereby, the mean image of PD patients derived from the PET-guided method shows higher uptake and in those regions. In contrast to the tendency shown in the mean images, the SD images of two CT-guided methods show higher SD values around the striatum, suggesting higher spatial variability. Voxel-based comparison between the controls and PD patients (family-wise error corrected) show the highest significance with the dtMR-guided method, and two CT-guided methods were inferior to the MR-guided methods. Color bars for mean and SD images represent standardized uptake value ratio (SUVR), and that for the voxel-based comparison images represents T-value for the comparison. Abbreviations: cvMR = MR-guided spatial normalization with conventional normalization tool, dtMR = MR-guided spatial normalization with DARTEL toolbox, ssCT = skull-stripped CT-guided spatial normalization, itCT = intensity transformed CT-guided spatial normalization, PET = PET-guided spatial normalization.

Mean SUVR images of the control subjects were similar across different spatial normalization methods. However, the mean SUVR image of the PET-guided method showed erroneous overestimation of the caudate and anterior putaminal uptake and lower contrast of asymmetry of putaminal uptake. Standard deviation (SD) images of dtMR- and PET-guided method in control subjects showed very low and rather homogeneous SD values in the entire striatum, whereas the SD images of the cvMR-guided and the two CT-guided methods showed higher SD values around the striatal border, suggesting greater spatial variability. As expected, the voxel-based comparison between the controls and PD patients showed the greatest differences in dtMR- and cvMR-guided methods (family-wise error corrected *P* < 0.001). Two CT-guided methods also showed highly significant differences across the entire putamen. Nevertheless, the level of significance for the anterior putamen was lowest for the PET-guided method (Fig 6).

## Discussion

This study found that two CT-guided spatial normalization methods are effective for the analysis of [^18^F]-FP-CIT PET images and almost comparable to the MR-guided methods in showing striatal dopaminergic deficit, in discriminating PD patients from controls, and in reflecting disease severity. Compared to the itCT-guided method, the striatal SUVR values derived from the ssCT-guided method showed slightly better correlation with those measured by FSVOI and with clinical severity measured by UPDRS motor score. The PET-guided method was not suitable for normalization of [^18^F]-FP-CIT PET images, because of its serious overestimation bias of striatal SUVR in PD patients.

Spatial normalization is crucial for unbiased template VOI-based or voxel-based analysis of PET images. Ligand-specific PET template-guided spatial normalization is easy to apply and has been used for various radioligands [2, 8, 20, 21]. Unlike the radioligands binding to the receptors distributed in widespread brain, the radioligands for dopamine transporter avidly bind to some restricted areas (e.g. striatum), and thus, spatial normalization is driven mainly by the striatum. The ligand-specific template for [^11^C]-raclopride PET, which shows similar binding pattern like dopamine transporter PET in healthy controls, works for spatial normalization and even better than the MR-guided method [2, 20]. In contrast, regional selectivity of nigral neuronal death in PD patients leads to selective loss of dopamine transporter binding to posterior putamen in dopamine transporter PET [4, 22]. Lack of dopamine transporter binding in the posterior putamen without structural loss and relative preservation of binding in the caudate and anterior putamen leads to spatial normalization error stretching the anterior putamen posteriorly and filling missing posterior putamen. Thereby, striatal binding can be extremely overestimated in PD patients compared to healthy controls as shown in this study. Striatal binding derived from PET-guided method may be less reflective of real striatal dopaminergic deficit and clinical severity. Nevertheless, ligand-specific template for presynaptic dopaminergic nerve terminal imaging had been used in several clinical studies without proper validation [19, 23, 24]. One study showed the feasibility of ligand-specific [^18^F]-FP-CIT PET template and good correlation between the striatal binding and clinical severity scores [25]. Since only the PD patients were analyzed, they might have missed the unbalanced overestimation of striatal binding between the PD patients and controls. To overcome this problem, summed image of entire time frames of dynamic PET, which represents delivery and specific binding of a radioligand [6], or perfusion-like PET derived from early time frames of dynamic PET can be used for the acquisition of spatial normalization parameter [2, 5, 26]. However, as longer scan time for the dynamic PET is necessary to get the summed image or early phase perfusion-like PET, it may not be suitable for routine clinical use.

MR images that provide structural information can increase the accuracy of spatial normalization of PET without being affected by the binding pattern of radioligands [7, 8], and this MR-guided method has been used by many clinical studies of PET. The accuracy can be more increased by removal of the skull and surrounding non-brain tissue [11–13], or by applying a deformation flow field acquired from different tissue types separately like DARTEL [16]. Structural images can also be provided by CT scanner although its resolution is much lower than the MR imaging. As the native CT images are inappropriate for spatial normalization, due to great differences in intensity between the skull and brain tissue, Rorden and colleagues developed a CT-guided spatial normalization method with intensity transformed CT image (itCT-guided method) [9]. They artificially modified intensity to increase the intensity range for brain tissue and to reduce the intensity of the skull. Another approach for CT-guided spatial normalization utilizes a skull-stripped CT [10]. They used Brain Extraction Tool (BET) for skull-stripping of CT image, but it is difficult to obtain optimal result with BET. We recently developed a sophisticated method for skull-stripping of CT image with SPM segmentation toolbox and created skull-stripped CT template for ssCT-guided spatial normalization [14]. As we expected, this ssCT-guided spatial normalization method for [^18^F]-FP-CIT PET showed almost the same result as the MR-guided methods for acquiring striatal SUVR values. Although there was no large difference between the performances of the ssCT- and itCT-guided methods, the ssCT-guided method showed slightly better correlation with the SUVR values measured with FSVOI (*P* < 0.05, Fisher’s r to z transformation) and with clinical severity scores.

Although our proposed ssCT-guided method can be a reliable and objective method for measuring striatal and even smaller subregional SUVR for [^18^F]-FP-CIT PET, the image processing steps are complicated and requires longer computing time [cvMR: 10 min, ssCT: 15–30 min, itCT: < 1 min, and PET: < 1min for single subject processing in Mac Pro early 2008 model (Apple Inc., Cupertino, CA) with a dual 3.2GHz quad core Xeon CPU and 16 GB memory]. The itCT-guided method has an advantage over the ssCT-guided method in that it is simpler and requires less computing time, although it was slightly less effective than the ssCT-guided method. Therefore, one of these CT-guided spatial normalization methods can be chosen, at least, for the analysis of [^18^F]-FP-CIT PET.

Internal subcortical gray matter structures cannot be visualized clearly in the CT images as the resolution of CT images is much lower than that of MR images. Therefore, it is clear that the MR-guided methods are best for the analysis of these subcortical structures and these CT-guided methods are alternatives, although we showed feasibility of these CT-guided methods in VOI-based analysis. This might have caused greater variability around the striatal border in the SD images derived from CT-guided methods (Fig 6).

Although we visually checked coregistration quality, another drawback common for the structure-guided methods is the possible misalignment in the coregistration step between the PET and structural images because of the very low uptake of [^18^F]-FP-CIT in the brain tissue surrounding the striatum. We neglected head motion during the acquisition of PET and did not coregistered PET to CT images due to short scan time. However, still there might be a possible head motion during this short scan time, and this may raise concern of bias in the SUVR values.

The advantage of these CT-guided spatial normalization methods for PET imaging is that they do not require additional structural images from a different scanner, because the PET/CT scanners typically provide structural CT images. Although the MR-guided methods are more accurate and the best for obtaining structural information, the CT-guided methods may be also useful when MR imaging is contraindicated due to an implanted cardiac pacemaker or claustrophobia and when a dynamic PET scan providing early phase perfusion-like PET images is unavailable. In addition, these methods may be useful for the PET imaging with various radioligands of which binding is limited to some regions of the brain or of which binding pattern is quite different between the controls and patients (e.g. [^11^C]-PIB). Future studies will be needed to validate these CT-guided methods for extension of these techniques to other types of radioligands.

## Conclusions

CT-guided spatial normalization methods provide reliable striatal SUVR values almost comparable to those obtained with MR-guided methods and can be useful for analyzing dopamine transporter PET images when structural MR images are unavailable.

## Supporting Information

S1 Fig Regression analysis between the SUVR values measured with individual volume of interest (FSVOI) and those derived from five different spatial normalization methods. Regression lines for each diagnostic group are presented with green (healthy controls; HC) and red (Parkinson’s disease; PD) dotted lines. Closed (HC) and open (PD) circles represent regional SUVR values of individual subjects. Regional SUVR values of control subjects were calculated as the mean of both sides. For PD patients the SUVR values were measured with the regional VOI contralateral to the clinically worse side (worse) and contralateral to the clinically better side (better). The R^2^ values for regression analysis in all subjects are presented in the right lower corner of each graph. The PET-guided spatial normalization method excessively overestimates striatal SUVR values in PD patients, and results in higher discrepancy in regression lines between the controls and PD patients.
(TIF)

S2 Fig Receiver-operator characteristic (ROC) curve analysis of striatal SUVR values derived from five different spatial normalization methods for the separation of controls and Parkinson’s disease (PD) patients. Data of the regions contralateral to the clinically worse (more affected side) and better sides (less affected side) were analyzed separately. The area under the curve (AUC) values are presented in the right lower corner of each graph. Putaminal SUVR values derived from all five spatial normalization methods very effectively discriminated PD patients, and those of the more affected side perform better than those of less affected side. Both CT-guided methods show similar performance to both MR-guided methods. However, the PET-guided method was less effective than the other four structure-guided spatial normalization methods.
(TIF)

S3 Fig Regression analysis between the striatal subregional SUVR values measured with individual volume of interest (FSVOI) and those derived from five different spatial normalization methods. Regression lines for each diagnostic group are presented with green (healthy controls; HC) and red (Parkinson’s disesase; PD) dotted lines. Closed (HC) and open (PD) circles represent regional SUVR values of individual subjects. Regional SUVR values of control subjects were calculated as the mean of both sides. For PD patients the SUVR values were measured with the regional VOI contralateral to the clinically worse side (worse) and contralateral to the clinically better side (better). The R^2^ values for regression analysis in all subjects are presented in the right lower corner of each graph. Although both MR-guided spatial normalization methods performed best for providing reliable subregional SUVR values, both CT-guided methods also worked almost similar to the cvMR-guided method. However, the PET-guided spatial normalization method excessively overestimated striatal SUVR values in PD patients, and resulted in higher discrepancy in regression lines between the controls and PD patients. This overestimation bias of the PET-guided method was most pronounced in the anterior putamen and the regions contralateral to the clinically more affected side.
(TIF)

S1 File Demographic data for the included subjects, raw data for the regional and subregional SUV and SUVR values, and the graphs created in this study. (ZIP)

S1 Table SUV values of striatal regions and cerebellum. Biases were calculated as the % difference between the SUV values derived from each spatial normalization method and those measured with FSVOI. For PD patients data are presented with the SUV values of combined region of both sides (both), those of the region contralateral to the clinically worse side (worse), and those of the region contralateral to the clinically better side (better). Abbreviations: FSVOI = FreeSurfer-generated volume of interest, cvMR = MR-guided spatial normalization with conventional tool, dtMR = MR-guided spatial normalization with DARTEL toolbox, ssCT = skull-stripped CT-guided spatial normalization, itCT = intensity transformed CT-guided spatial normalization, PET = PET-guided spatial normalization.
(DOC)

S2 Table Statistical comparison between the SUVR values derived from each spatial normalization method and those measured with FSVOI. The statistical significance of independent t-test between the SUVR values derived from each spatial normalization method and those measured with FSVOI are presented with *P*-values for each comparison. For PD patients *P*-values are presented with those of combined region of both sides (both), those of the region contralateral to the clinically worse side (worse), and those of the region contralateral to the clinically better side (better). Abbreviations: FSVOI = FreeSurfer-generated volume of interest, cvMR = MR-guided spatial normalization with conventional tool, dtMR = MR-guided spatial normalization with DARTEL toolbox, ssCT = skull-stripped CT-guided spatial normalization, itCT = intensity transformed CT-guided spatial normalization, PET = PET-guided spatial normalization, n.s. = not significant.
(DOC)

## References

[1] Ravina B, Eidelberg D, Ahlskog JE, Albin RL, Brooks DJ, Carbon M, et al The role of radiotracer imaging in Parkinson disease. Neurology. 2005;64:208–215. 15668415 10.1212/01.WNL.0000149403.14458.7F

[2] Kuhn FP, Warnock GI, Burger C, Ledermann K, Martin-Soelch C, Buck A. Comparison of PET template-based and MRI-based image processing in the quantitative analysis of C11-raclopride PET. EJNMMI Res. 2014;4:7 10.1186/2191-219X-4-7 24451009 PMC3904930

[3] Yasuno F, Hasnine AH, Suhara T, Ichimiya T, Sudo Y, Inoue M, et al Template-based method for multiple volumes of interest of human brain PET images. Neuroimage. 2002;16:577–586. 12169244 10.1006/nimg.2002.1120

[4] Brooks DJ. Imaging approaches to Parkinson disease. J Nucl Med. 2010;51:596–609. 10.2967/jnumed.108.059998 20351351

[5] Ma Y, Dhawan V, Mentis M, Chaly T, Spetsieris PG, Eidelberg D. Parametric mapping of [18F]FPCIT binding in early stage Parkinson's disease: a PET study. Synapse. 2002;45:125–133. 12112405 10.1002/syn.10090

[6] Pavese N, Rivero-Bosch M, Lewis SJ, Whone AL, Brooks DJ. Progression of monoaminergic dysfunction in Parkinson's disease: a longitudinal 18F-dopa PET study. Neuroimage. 2011;56:1463–1468. 10.1016/j.neuroimage.2011.03.012 21396455

[7] Ashburner J, Friston KJ. Nonlinear spatial normalization using basis functions. Hum Brain Mapp. 1999;7:254–266. 10408769 10.1002/(SICI)1097-0193(1999)7:4<254::AID-HBM4>3.0.CO;2-G PMC6873340

[8] Gispert JD, Pascau J, Reig S, Martinez-Lazaro R, Molina V, Garcia-Barreno P, et al Influence of the normalization template on the outcome of statistical parametric mapping of PET scans. Neuroimage. 2003;19:601–612. 12880791 10.1016/s1053-8119(03)00072-7

[9] Rorden C, Bonilha L, Fridriksson J, Bender B, Karnath HO. Age-specific CT and MRI templates for spatial normalization. Neuroimage. 2012;61:957–965. 10.1016/j.neuroimage.2012.03.020 22440645 PMC3376197

[10] Solomon J, Raymont V, Braun A, Butman JA, Grafman J. User-friendly software for the analysis of brain lesions (ABLe). Comput Methods Programs Biomed. 2007;86:245–254. 17408802 10.1016/j.cmpb.2007.02.006 PMC1995425

[11] Acosta-Cabronero J, Williams GB, Pereira JM, Pengas G, Nestor PJ. The impact of skull-stripping and radio-frequency bias correction on grey-matter segmentation for voxel-based morphometry. Neuroimage. 2008;39:1654–1665. 18065243 10.1016/j.neuroimage.2007.10.051

[12] Fein G, Landman B, Tran H, Barakos J, Moon K, Di Sclafani V, et al Statistical parametric mapping of brain morphology: sensitivity is dramatically increased by using brain-extracted images as inputs. Neuroimage. 2006;30:1187–1195. 16442817 10.1016/j.neuroimage.2005.10.054 PMC1987363

[13] Fischmeister FP, Hollinger I, Klinger N, Geissler A, Wurnig MC, Matt E, et al The benefits of skull stripping in the normalization of clinical fMRI data. Neuroimage Clin. 2013;3:369–380. 10.1016/j.nicl.2013.09.007 24273720 PMC3814956

[14] Cho H, Kim JS, Choi JY, Ryu YH, Lyoo CH. A Computed Tomography-Based Spatial Normalization for the Analysis of [18F] Fluorodeoxyglucose Positron Emission Tomography of the Brain. Korean J Radiol. 2014;15:862–870. 10.3348/kjr.2014.15.6.862 25469101 PMC4248645

[15] Hughes AJ, Daniel SE, Kilford L, Lees AJ. Accuracy of clinical diagnosis of idiopathic Parkinson's disease: a clinico-pathological study of 100 cases. J Neurol Neurosurg Psychiatry. 1992;55:181–184. 1564476 10.1136/jnnp.55.3.181 PMC1014720

[16] Ashburner J. A fast diffeomorphic image registration algorithm. Neuroimage. 2007;38:95–113. 17761438 10.1016/j.neuroimage.2007.07.007

[17] Fischl B, Salat DH, Busa E, Albert M, Dieterich M, Haselgrove C, et al Whole brain segmentation: automated labeling of neuroanatomical structures in the human brain. Neuron. 2002;33:341–355. 11832223 10.1016/s0896-6273(02)00569-x

[18] Mawlawi O, Martinez D, Slifstein M, Broft A, Chatterjee R, Hwang DR, et al Imaging human mesolimbic dopamine transmission with positron emission tomography: I. Accuracy and precision of D2 receptor parameter measurements in ventral striatum. J Cereb Blood Flow Metab. 2001;21:1034–1057. 11524609 10.1097/00004647-200109000-00002

[19] Oh M, Kim JS, Kim JY, Shin KH, Park SH, Kim HO, et al Subregional patterns of preferential striatal dopamine transporter loss differ in Parkinson disease, progressive supranuclear palsy, and multiple-system atrophy. J Nucl Med. 2012;53:399–406. 10.2967/jnumed.111.095224 22323779

[20] Meyer JH, Gunn RN, Myers R, Grasby PM. Assessment of spatial normalization of PET ligand images using ligand-specific templates. Neuroimage. 1999;9:545–553. 10329294 10.1006/nimg.1999.0431

[21] Edison P, Carter SF, Rinne JO, Gelosa G, Herholz K, Nordberg A, et al Comparison of MRI based and PET template based approaches in the quantitative analysis of amyloid imaging with PIB-PET. Neuroimage. 2013;70:423–433. 10.1016/j.neuroimage.2012.12.014 23261639

[22] Fearnley JM, Lees AJ. Ageing and Parkinson's disease: substantia nigra regional selectivity. Brain. 1991;114:2283–2301. 1933245 10.1093/brain/114.5.2283

[23] Eggers C, Schwartz F, Pedrosa DJ, Kracht L, Timmermann L. Parkinson's disease subtypes show a specific link between dopaminergic and glucose metabolism in the striatum. PLoS One. 2014;9:e96629 10.1371/journal.pone.0096629 24848641 PMC4029550

[24] Lyoo CH, Ryu YH, Lee MJ, Lee MS. Striatal dopamine loss and discriminative sensory dysfunction in Parkinson's disease. Acta Neurol Scand. 2012;126:344–349. 10.1111/j.1600-0404.2012.01657.x 22380639

[25] Jeong E, Oh SY, Pahk K, Lee CN, Park KW, Lee JS, et al Feasibility of PET Template-Based Analysis on F-18 FP-CIT PET in Patients with De Novo Parkinson's Disease. Nucl Med Mol Imaging. 2013;47:73–80. 10.1007/s13139-013-0196-6 24900086 PMC4041974

[26] Rostomian AH, Madison C, Rabinovici GD, Jagust WJ. Early 11C-PIB frames and 18F-FDG PET measures are comparable: a study validated in a cohort of AD and FTLD patients. J Nucl Med. 2011;52:173–179. 10.2967/jnumed.110.082057 21233181 PMC3166243

