# Supplementary figures and images for: Feasibility of Computed Tomography-Guided Methods for Spatial Normalization of Dopamine Transporter Positron Emission Tomography Image
Source: PLoS One. 2015 Jul 6;10(7):e0132585. doi: 10.1371/journal.pone.0132585 (PMC4492980; doi:10.1371/journal.pone.0132585)

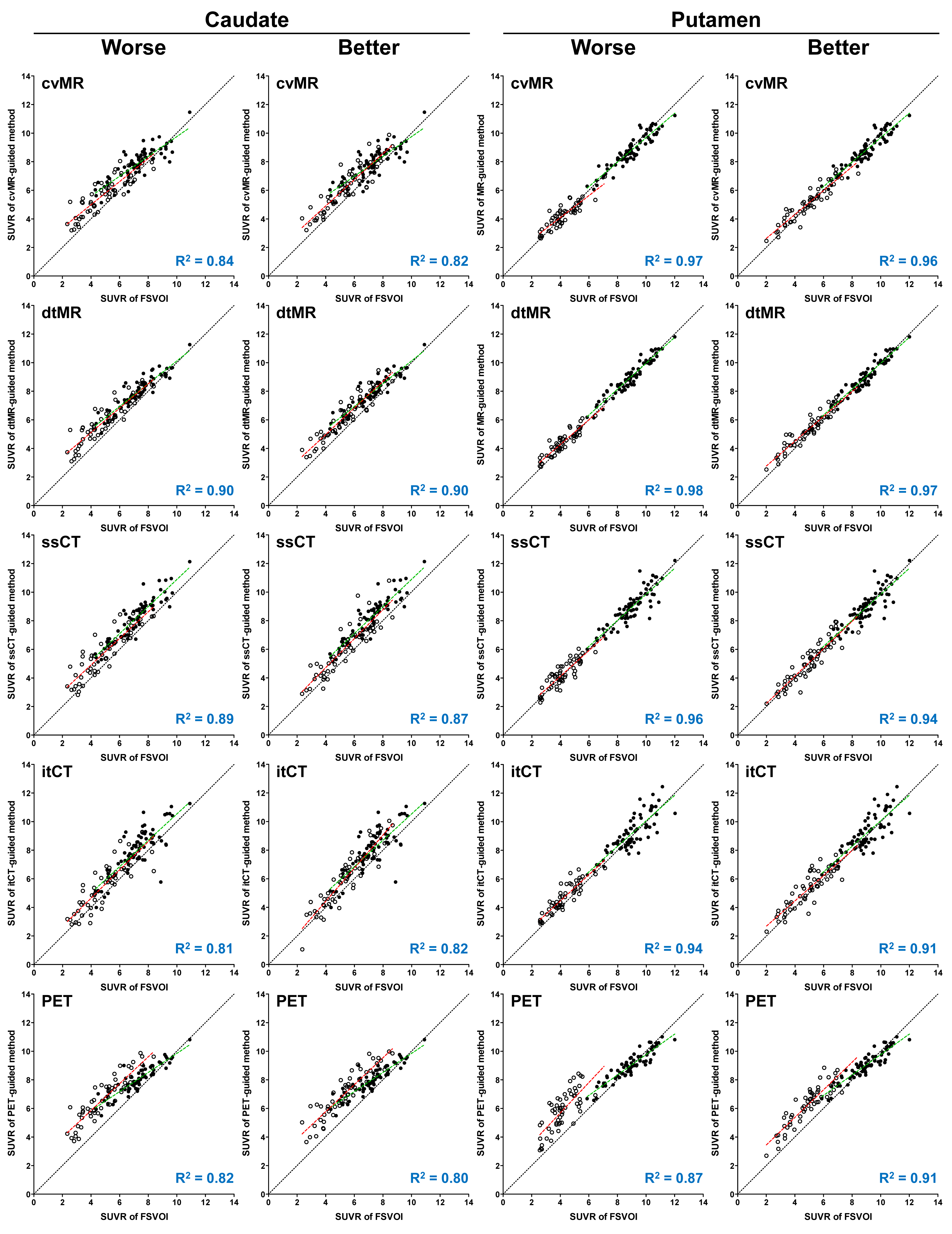

Supplement: S1 Fig — Regression lines for each diagnostic group are presented with green (healthy controls; HC) and red (Parkinson’s disease; PD) dotted lines. Closed (HC) and open (PD) circles represent regional SUVR values of individual subjects. Regional SUVR values of control subjects were calculated as the mean of both sides. For PD patients the SUVR values were measured with the regional VOI contralateral to the clinically worse side (worse) and contralateral to the clinically better side (better). The R2 values for regression analysis in all subjects are presented in the right lower corner of each graph. The PET-guided spatial normalization method excessively overestimates striatal SUVR values in PD patients, and results in higher discrepancy in regression lines between the controls and PD patients. (TIF) [file pone.0132585.s001.tif]

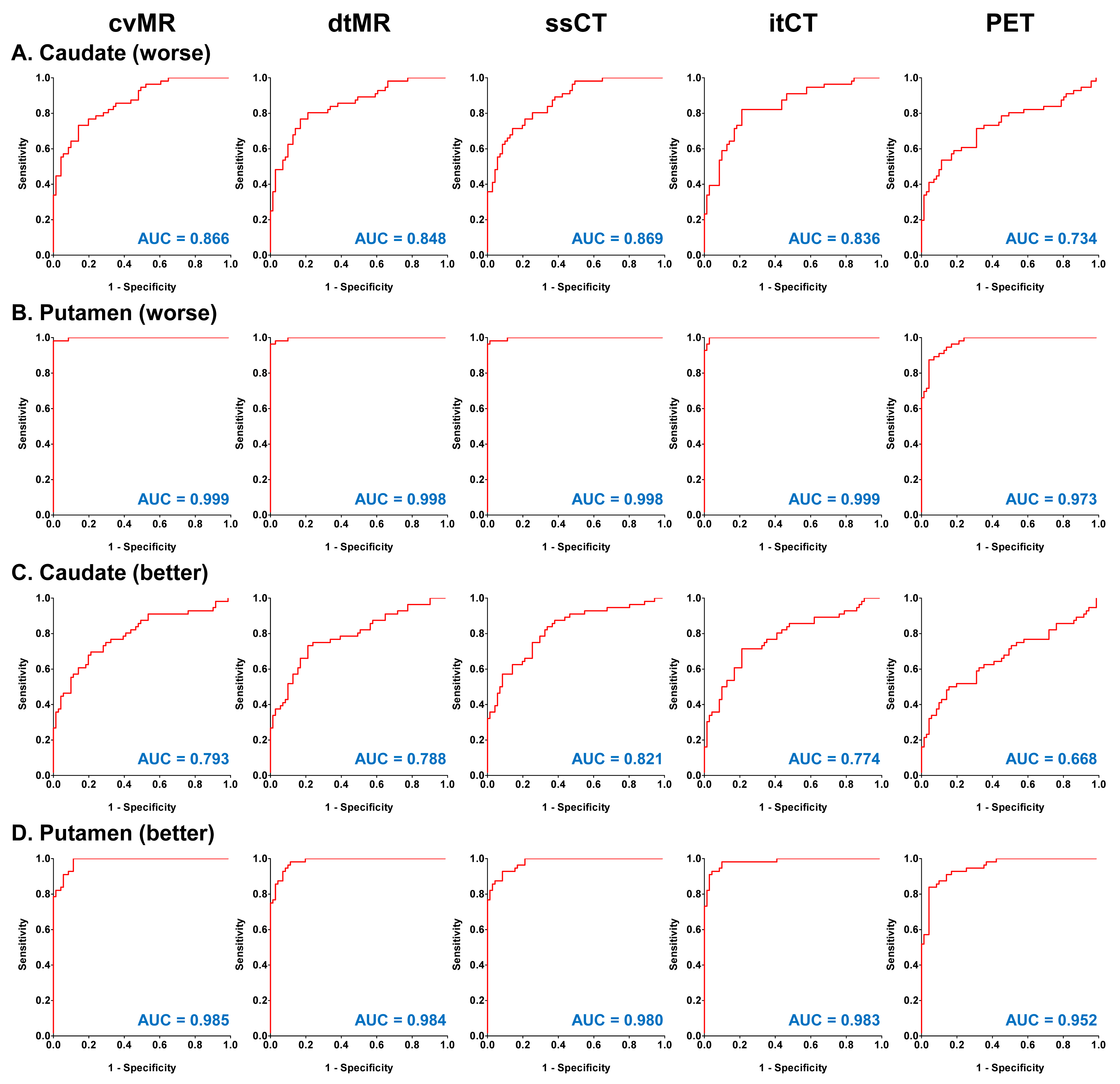

Supplement: S2 Fig — Data of the regions contralateral to the clinically worse (more affected side) and better sides (less affected side) were analyzed separately. The area under the curve (AUC) values are presented in the right lower corner of each graph. Putaminal SUVR values derived from all five spatial normalization methods very effectively discriminated PD patients, and those of the more affected side perform better than those of less affected side. Both CT-guided methods show similar performance to both MR-guided methods. However, the PET-guided method was less effective than the other four structure-guided spatial normalization methods. (TIF) [file pone.0132585.s002.tif]

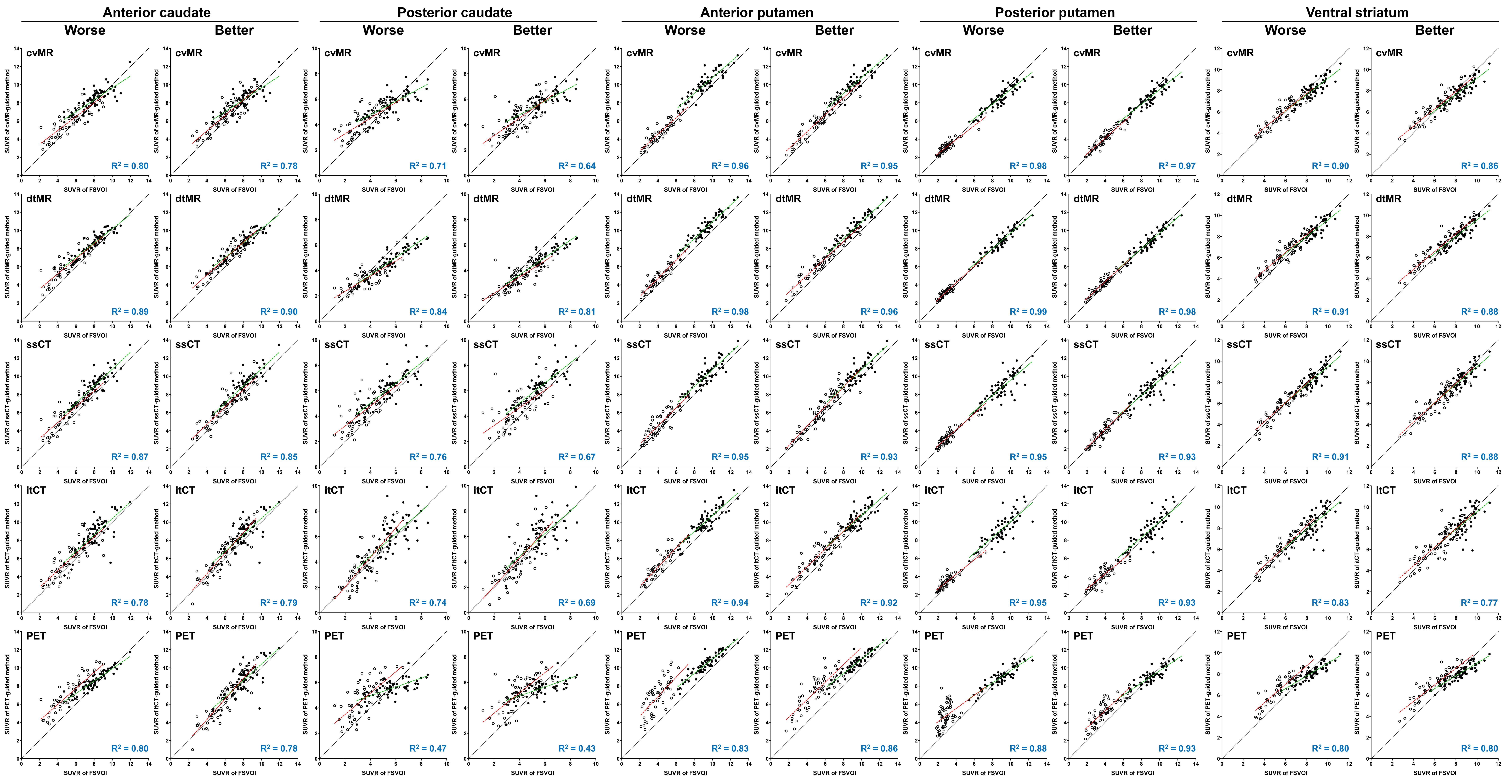

Supplement: S3 Fig — Regression lines for each diagnostic group are presented with green (healthy controls; HC) and red (Parkinson’s disesase; PD) dotted lines. Closed (HC) and open (PD) circles represent regional SUVR values of individual subjects. Regional SUVR values of control subjects were calculated as the mean of both sides. For PD patients the SUVR values were measured with the regional VOI contralateral to the clinically worse side (worse) and contralateral to the clinically better side (better). The R2 values for regression analysis in all subjects are presented in the right lower corner of each graph. Although both MR-guided spatial normalization methods performed best for providing reliable subregional SUVR values, both CT-guided methods also worked almost similar to the cvMR-guided method. However, the PET-guided spatial normalization method excessively overestimated striatal SUVR values in PD patients, and resulted in higher discrepancy in regression lines between the controls and PD patients. This overestimation bias of the PET-guided method was most pronounced in the anterior putamen and the regions contralateral to the clinically more affected side. (TIF) [file pone.0132585.s003.tif]
