# Supplementary material for: Feasibility of Computed Tomography-Guided Methods for Spatial Normalization of Dopamine Transporter Positron Emission Tomography Image
Source: PLoS One. 2015 Jul 6;10(7):e0132585. doi: 10.1371/journal.pone.0132585 (PMC4492980; doi:10.1371/journal.pone.0132585)
Supplement: S1 Table — Biases were calculated as the % difference between the SUV values derived from each spatial normalization method and those measured with FSVOI. For PD patients data are presented with the SUV values of combined region of both sides (both), those of the region contralateral to the clinically worse side (worse), and those of the region contralateral to the clinically better side (better). Abbreviations: FSVOI = FreeSurfer-generated volume of interest, cvMR = MR-guided spatial normalization with conventional tool, dtMR = MR-guided spatial normalization with DARTEL toolbox, ssCT = skull-stripped CT-guided spatial normalization, itCT = intensity transformed CT-guided spatial normalization, PET = PET-guided spatial normalization. (DOC) [file pone.0132585.s005.doc]

**S1 Table.** SUV values of striatal regions and cerebellum

|  | | **FSVOI** | | |  | **cvMR** | | | |  | **dtMR** | | | |  | **ssCT** | | | |  | **itCT** | | | |  | **PT** | | | |
| --- | --- | --- | --- | --- | --- | --- | --- | --- | --- | --- | --- | --- | --- | --- | --- | --- | --- | --- | --- | --- | --- | --- | --- | --- | --- | --- | --- | --- | --- |
|  | | **mean** | **±** | **SD** |  | **mean** | **±** | **SD** | **bias** |  | **mean** | **±** | **SD** | **bias** |  | **mean** | **±** | **SD** | **bias** |  | **mean** | **±** | **SD** | **bias** |  | **mean** | **±** | **SD** | **bias** |
| **Caudate** | | | | | | | | | | | | | | | | | | | | | | | | | | | | | |
| **HC** | **both** | 1.50 | ± | 0.38 |  | 1.65 | ± | 0.36 | 10.6 |  | 1.62 | ± | 0.36 | 8.1 |  | 1.74 | ± | 0.41 | 16.2 |  | 1.67 | ± | 0.42 | 11.4 |  | 1.68 | ± | 0.36 | 12.2 |
| **PD** | **both** | 1.20 | ± | 0.50 |  | 1.40 | ± | 0.50 | 16.6 |  | 1.38 | ± | 0.50 | 15.0 |  | 1.40 | ± | 0.54 | 16.5 |  | 1.36 | ± | 0.54 | 12.8 |  | 1.59 | ± | 0.54 | 32.6 |
|  | **worse** | 1.15 | ± | 0.50 |  | 1.35 | ± | 0.48 | 17.7 |  | 1.34 | ± | 0.49 | 16.9 |  | 1.36 | ± | 0.51 | 18.2 |  | 1.30 | ± | 0.53 | 13.3 |  | 1.56 | ± | 0.54 | 35.3 |
|  | **better** | 1.25 | ± | 0.52 |  | 1.45 | ± | 0.53 | 15.9 |  | 1.42 | ± | 0.53 | 13.6 |  | 1.44 | ± | 0.57 | 15.1 |  | 1.41 | ± | 0.59 | 12.7 |  | 1.63 | ± | 0.55 | 30.4 |
| **Anterior caudate** | | | | | | | | | | | | | | | | | | | | | | | | | | | | | |
| **HC** | **both** | 1.63 | ± | 0.41 |  | 1.75 | ± | 0.39 | 6.9 |  | 1.76 | ± | 0.40 | 7.6 |  | 1.86 | ± | 0.44 | 13.7 |  | 1.78 | ± | 0.46 | 8.7 |  | 1.78 | ± | 0.39 | 8.9 |
| **PD** | **both** | 1.32 | ± | 0.57 |  | 1.47 | ± | 0.54 | 11.9 |  | 1.50 | ± | 0.56 | 13.8 |  | 1.49 | ± | 0.58 | 13.1 |  | 1.45 | ± | 0.59 | 10.1 |  | 1.70 | ± | 0.59 | 29.3 |
|  | **worse** | 1.25 | ± | 0.56 |  | 1.41 | ± | 0.52 | 12.9 |  | 1.45 | ± | 0.54 | 15.6 |  | 1.44 | ± | 0.55 | 15.0 |  | 1.39 | ± | 0.56 | 10.7 |  | 1.66 | ± | 0.58 | 32.6 |
|  | **better** | 1.38 | ± | 0.60 |  | 1.53 | ± | 0.57 | 11.3 |  | 1.55 | ± | 0.59 | 12.6 |  | 1.54 | ± | 0.63 | 11.7 |  | 1.51 | ± | 0.66 | 9.9 |  | 1.74 | ± | 0.59 | 26.7 |
| **Posterior caudate** | | | | | | | | | | | | | | | | | | | | | | | | | | | | | |
| **HC** | **both** | 1.14 | ± | 0.34 |  | 1.18 | ± | 0.28 | 3.6 |  | 0.91 | ± | 0.27 | -19.8 |  | 1.31 | ± | 0.33 | 15.0 |  | 1.21 | ± | 0.39 | 6.1 |  | 1.16 | ± | 0.27 | 1.2 |
| **PD** | **both** | 0.88 | ± | 0.35 |  | 1.02 | ± | 0.38 | 16.1 |  | 0.76 | ± | 0.28 | -13.8 |  | 1.06 | ± | 0.41 | 20.7 |  | 0.94 | ± | 0.43 | 7.1 |  | 1.15 | ± | 0.43 | 30.7 |
|  | **worse** | 0.82 | ± | 0.36 |  | 0.99 | ± | 0.36 | 21.1 |  | 0.74 | ± | 0.28 | -9.6 |  | 1.04 | ± | 0.39 | 26.5 |  | 0.89 | ± | 0.44 | 8.2 |  | 1.10 | ± | 0.43 | 34.2 |
|  | **better** | 0.91 | ± | 0.39 |  | 1.06 | ± | 0.41 | 15.9 |  | 0.78 | ± | 0.31 | -14.6 |  | 1.09 | ± | 0.45 | 19.4 |  | 0.99 | ± | 0.47 | 8.2 |  | 1.18 | ± | 0.43 | 29.9 |
| **Putamen** | | | | | | | | | | | | | | | | | | | | | | | | | | | | | |
| **HC** | **both** | 1.86 | ± | 0.40 |  | 1.86 | ± | 0.40 | 0.1 |  | 1.90 | ± | 0.41 | 2.6 |  | 1.88 | ± | 0.41 | 1.2 |  | 1.91 | ± | 0.41 | 3.1 |  | 1.89 | ± | 0.38 | 2.0 |
| **PD** | **both** | 1.06 | ± | 0.41 |  | 1.10 | ± | 0.40 | 3.6 |  | 1.11 | ± | 0.40 | 4.5 |  | 1.10 | ± | 0.42 | 3.9 |  | 1.16 | ± | 0.42 | 9.5 |  | 1.41 | ± | 0.50 | 32.3 |
|  | **worse** | 0.97 | ± | 0.37 |  | 1.00 | ± | 0.33 | 2.3 |  | 1.01 | ± | 0.35 | 3.4 |  | 1.01 | ± | 0.36 | 3.9 |  | 1.09 | ± | 0.37 | 11.4 |  | 1.36 | ± | 0.48 | 39.6 |
|  | **better** | 1.15 | ± | 0.50 |  | 1.20 | ± | 0.48 | 4.3 |  | 1.21 | ± | 0.48 | 5.2 |  | 1.19 | ± | 0.51 | 3.4 |  | 1.24 | ± | 0.50 | 7.6 |  | 1.45 | ± | 0.52 | 25.7 |
| **Anterior putamen** | | | | | | | | | | | | | | | | | | | | | | | | | | | | | |
| **HC** | **both** | 1.92 | ± | 0.42 |  | 2.14 | ± | 0.46 | 11.7 |  | 2.14 | ± | 0.46 | 11.7 |  | 2.12 | ± | 0.46 | 10.5 |  | 2.11 | ± | 0.45 | 10.1 |  | 2.18 | ± | 0.45 | 13.6 |
| **PD** | **both** | 1.07 | ± | 0.44 |  | 1.23 | ± | 0.47 | 14.9 |  | 1.23 | ± | 0.48 | 15.5 |  | 1.22 | ± | 0.51 | 14.2 |  | 1.32 | ± | 0.51 | 23.8 |  | 1.67 | ± | 0.61 | 56.2 |
|  | **worse** | 0.95 | ± | 0.38 |  | 1.09 | ± | 0.40 | 14.1 |  | 1.10 | ± | 0.41 | 15.0 |  | 1.10 | ± | 0.44 | 15.3 |  | 1.21 | ± | 0.44 | 27.0 |  | 1.61 | ± | 0.59 | 68.5 |
|  | **better** | 1.18 | ± | 0.56 |  | 1.37 | ± | 0.58 | 15.8 |  | 1.38 | ± | 0.58 | 16.4 |  | 1.34 | ± | 0.65 | 13.6 |  | 1.44 | ± | 0.63 | 21.7 |  | 1.73 | ± | 0.65 | 46.7 |
| **Posterior putamen** | | | | | | | | | | | | | | | | | | | | | | | | | | | | | |
| **HC** | **both** | 1.86 | ± | 0.42 |  | 1.82 | ± | 0.40 | -1.8 |  | 1.92 | ± | 0.42 | 3.7 |  | 1.83 | ± | 0.43 | -1.2 |  | 1.91 | ± | 0.43 | 2.9 |  | 1.88 | ± | 0.38 | 1.4 |
| **PD** | **both** | 0.79 | ± | 0.35 |  | 0.85 | ± | 0.36 | 6.6 |  | 0.86 | ± | 0.37 | 8.2 |  | 0.85 | ± | 0.38 | 7.5 |  | 0.89 | ± | 0.36 | 12.7 |  | 1.15 | ± | 0.45 | 44.7 |
|  | **worse** | 0.70 | ± | 0.33 |  | 0.75 | ± | 0.31 | 7.0 |  | 0.75 | ± | 0.33 | 7.7 |  | 0.76 | ± | 0.34 | 9.0 |  | 0.81 | ± | 0.32 | 15.4 |  | 1.11 | ± | 0.44 | 58.7 |
|  | **better** | 0.90 | ± | 0.44 |  | 0.95 | ± | 0.45 | 5.3 |  | 0.97 | ± | 0.45 | 7.5 |  | 0.95 | ± | 0.46 | 5.3 |  | 0.98 | ± | 0.44 | 9.4 |  | 1.19 | ± | 0.48 | 32.4 |
| **Ventral striatum** | | | | | | | | | | | | | | | | | | | | | | | | | | | | | |
| **HC** | **both** | 1.76 | ± | 0.38 |  | 1.69 | ± | 0.36 | -3.8 |  | 1.70 | ± | 0.36 | -3.3 |  | 1.73 | ± | 0.37 | -1.6 |  | 1.74 | ± | 0.40 | -1.3 |  | 1.70 | ± | 0.33 | -3.3 |
| **PD** | **both** | 1.37 | ± | 0.52 |  | 1.44 | ± | 0.47 | 4.9 |  | 1.46 | ± | 0.47 | 6.6 |  | 1.41 | ± | 0.49 | 2.9 |  | 1.44 | ± | 0.51 | 5.3 |  | 1.59 | ± | 0.50 | 16.2 |
|  | **worse** | 1.32 | ± | 0.48 |  | 1.37 | ± | 0.44 | 4.2 |  | 1.40 | ± | 0.45 | 6.8 |  | 1.36 | ± | 0.46 | 3.2 |  | 1.41 | ± | 0.49 | 7.1 |  | 1.55 | ± | 0.50 | 18.0 |
|  | **better** | 1.43 | ± | 0.56 |  | 1.50 | ± | 0.51 | 5.1 |  | 1.51 | ± | 0.51 | 6.0 |  | 1.46 | ± | 0.54 | 2.2 |  | 1.48 | ± | 0.54 | 3.2 |  | 1.63 | ± | 0.51 | 14.0 |
| **Cerebellum** | | | | | | | | | | | | | | | | | | | | | | | | | | | | | |
| **HC** | **both** | 0.21 | ± | 0.05 |  | 0.21 | ± | 0.05 | 1.9 |  | 0.21 | ± | 0.04 | -2.0 |  | 0.21 | ± | 0.05 | 1.8 |  | 0.21 | ± | 0.05 | 0.9 |  | 0.21 | ± | 0.05 | 1.1 |
| **PD** | **both** | 0.23 | ± | 0.06 |  | 0.23 | ± | 0.06 | 1.6 |  | 0.23 | ± | 0.06 | -2.0 |  | 0.24 | ± | 0.06 | 1.8 |  | 0.23 | ± | 0.06 | 1.2 |  | 0.23 | ± | 0.06 | -0.1 |

Biases were calculated as the % difference between the SUV values derived from each spatial normalization method and those measured with FSVOI. For PD patients data are presented with the SUV values of combined region of both sides (both), those of the region contralateral to the clinically worse side (worse), and those of the region contralateral to the clinically better side (better).

Abbreviations: FSVOI = FreeSurfer-generated volume of interest, cvMR = MR-guided spatial normalization with conventional tool, dtMR = MR-guided spatial normalization with DARTEL toolbox, ssCT = skull-stripped CT-guided spatial normalization, itCT = intensity transformed CT-guided spatial normalization, PET = PET-guided spatial normalization
