# Supplementary material for: Feasibility of Computed Tomography-Guided Methods for Spatial Normalization of Dopamine Transporter Positron Emission Tomography Image
Source: PLoS One. 2015 Jul 6;10(7):e0132585. doi: 10.1371/journal.pone.0132585 (PMC4492980; doi:10.1371/journal.pone.0132585)
Supplement: S2 Table — The statistical significance of independent t-test between the SUVR values derived from each spatial normalization method and those measured with FSVOI are presented with P-values for each comparison. For PD patients P-values are presented with those of combined region of both sides (both), those of the region contralateral to the clinically worse side (worse), and those of the region contralateral to the clinically better side (better). Abbreviations: FSVOI = FreeSurfer-generated volume of interest, cvMR = MR-guided spatial normalization with conventional tool, dtMR = MR-guided spatial normalization with DARTEL toolbox, ssCT = skull-stripped CT-guided spatial normalization, itCT = intensity transformed CT-guided spatial normalization, PET = PET-guided spatial normalization, n.s. = not significant. (DOC) [file pone.0132585.s006.doc]

**S2 Table.** Statistical comparison between the SUVR values derived from each spatial normalization method and those measured with FSVOI

|  | | | | | | **cvMR** | **dtMR** | **ssCT** | **itCT** | **PET** |
| --- | --- | --- | --- | --- | --- | --- | --- | --- | --- | --- |
| **Caudate** | | | | | | | | | | |
| **HC** | | | | | **both** | 0.005 | 0.002 | 2.7×10^-5^ | 0.003 | 2.3×10^-4^ |
| **PD** | | | | | **both** | 0.006 | 0.002 | 0.011 | 0.043 | 4.2×10^-8^ |
|  | | | | | **worse** | 0.005 | 0.001 | 0.007 | 0.045 | 2.8×10^-8^ |
|  | | | | | **better** | 0.010 | 0.004 | 0.023 | n.s. | 1.5×10^-7^ |
| **Anterior caudate** | | | | | | | | | | |
| **HC** | | | | **both** | | n.s. | 0.008 | 0.001 | 0.026 | 0.011 |
| **PD** | | | | **both** | | n.s. | 0.005 | n.s. | n.s. | 1.0×10^-6^ |
|  | | | | **worse** | | n.s. | 0.003 | 0.034 | n.s. | 5.2×10^-7^ |
|  | | | | **better** | | n.s. | 0.009 | n.s. | n.s. | 4.0×10^-6^ |
| **Posterior caudate** | | | | | | | | | | |
| **HC** | | | | **both** | | n.s. | 1.2×10^-4^ | 0.002 | n.s. | n.s. |
| **PD** | | | | **both** | | 0.011 | 0.017 | 0.002 | n.s. | 1.2×10^-6^ |
|  | | | | **worse** | | 0.002 | n.s. | 2.9×10^-4^ | n.s. | 3.1×10^-6^ |
|  | | | | **better** | | 0.024 | 0.021 | 0.008 | n.s. | 4.1×10^-6^ |
| **Putamen** | | | | | | | | | | |
| **HC** | | | **both** | | | n.s. | n.s. | n.s. | n.s. | n.s. |
| **PD** | | | **both** | | | n.s. | n.s. | n.s. | 0.047 | 2.1×10^-9^ |
|  | | | **worse** | | | n.s. | n.s. | n.s. | 0.020 | 2.4×10^-11^ |
|  | | | **better** | | | n.s. | n.s. | n.s. | n.s. | 1.9×10^-6^ |
| **Anterior putamen** | | | | | | | | | | |
| **HC** | | **both** | | | | 3.2×10^-4^ | 5.3×10^-5^ | 0.001 | 0.001 | 1.5×10^-6^ |
| **PD** | | **both** | | | | 0.015 | 0.001 | 0.036 | 1.1×10^-4^ | 1.6×10^-14^ |
|  | | **worse** | | | | 0.026 | 0.003 | 0.028 | 3.7×10^-5^ | 6.1×10^-17^ |
|  | | **better** | | | | 0.022 | 0.003 | n.s. | 0.002 | 1.1×10^-10^ |
| **Posterior putamen** | | | | | | | | | | |
| **HC** | **both** | | | | | n.s. | n.s. | n.s. | n.s. | n.s. |
| **PD** | **both** | | | | | n.s. | 0.041 | n.s. | 0.016 | 1.7×10^-11^ |
|  | **worse** | | | | | n.s. | n.s. | n.s. | 0.012 | 5.2×10^-13^ |
|  | **better** | | | | | n.s. | n.s. | n.s. | n.s. | 2.3×10^-7^ |
| **Ventral striatum** | | | | | | | | | | |
| **HC** | | **both** | | | | 0.009 | n.s. | n.s. | n.s. | 0.026 |
| **PD** | | **both** | | | | n.s. | 0.042 | n.s. | n.s. | 3.0×10^-4^ |
|  | | **worse** | | | | n.s. | 0.041 | n.s. | n.s. | 1.2×10^-4^ |
|  | | **better** | | | | n.s. | n.s. | n.s. | n.s. | 0.001 |

The statistical significance of independent t-test between the SUVR values derived from each spatial normalization method and those measured with FSVOI are presented with *P*-values for each comparison. For PD patients *P*-values are presented with those of combined region of both sides (both), those of the region contralateral to the clinically worse side (worse), and those of the region contralateral to the clinically better side (better).

Abbreviations: FSVOI = FreeSurfer-generated volume of interest, cvMR = MR-guided spatial normalization with conventional tool, dtMR = MR-guided spatial normalization with DARTEL toolbox, ssCT = skull-stripped CT-guided spatial normalization, itCT = intensity transformed CT-guided spatial normalization, PET = PET-guided spatial normalization, n.s. = not significant
